# Antifungal Activity of Denture Base Resin Containing Nanozirconia: In Vitro Assessment of *Candida albicans* Biofilm

**DOI:** 10.1155/2021/5556413

**Published:** 2021-07-31

**Authors:** Reem Abualsaud, Doaa M. Aleraky, Sultan Akhtar, Soban Q. Khan, Mohammed M. Gad

**Affiliations:** ^1^Department of Substitutive Dental Sciences, College of Dentistry, Imam Abdulrahman Bin Faisal University, P.O. Box 1982, Dammam 31441, Saudi Arabia; ^2^Department of Biomedical Dental Sciences, College of Dentistry, Imam Abdulrahman Bin Faisal University, P.O. Box 1982, Dammam 31441, Saudi Arabia; ^3^Department of Biophysics, Institute for Research and Medical Consultations, Imam Abdulrahman Bin Faisal University, P.O. Box 1982, Dammam 31441, Saudi Arabia; ^4^Department of Dental Education, College of Dentistry, Imam Abdulrahman Bin Faisal University, P.O. Box 1982, Dammam 31441, Saudi Arabia

## Abstract

**Objective:**

To evaluate the antimicrobial effects of different concentrations of zirconium dioxide nanoparticles (nano-ZrO_2_) reinforcement of poly(methyl) methacrylate (PMMA) on surface roughness and *C. albicans* biofilm.

**Methods:**

20 heat-polymerized acrylic resin discs were conventionally made and divided into 4 groups (*n* = 5) according to nano-ZrO_2_ concentration: control (0% filler) and 3 experimental groups (2.5% (Z2.5), 5.0% (Z5.0), and 7.5% (Z7.5)). An optical profilometer was used for surface roughness evaluation, followed by *Candida* adherence assay. Specimens were sterilized, then immersed in cultured yeast (*C. albicans*), and incubated at 37°C for 48 hours. After that, discs were rinsed before extracting the clustered pellets of *Candida.* The attached *C. albicans* was counted using the direct method after spreading on agar media and incubating for 48 hours. Statistical analysis was performed using one-way ANOVA and Tukey's post hoc test at *α* = 0.05.

**Results:**

Surface roughness was significantly increased with all modified groups compared with control (*P* < 0.01), which showed the lowest roughness value (0.027 ± 0.004 *μ*m). There was no significant difference in the roughness value among reinforced groups (2.5, 5.0, and 7.5%) (*P* > 0.05), with Z7.5 showing the highest roughness value (0.042 ± 0.004 *μ*m). *Candida* count was reduced as the nano-ZrO_2_ increased but not significantly (*P*=0.15).

**Conclusions:**

The addition of different concentrations of nano-ZrO_2_ particles to PMMA increased the surface roughness compared with control; in contrast, insignificant reduction of *C. albicans* biofilm was detected.

## 1. Introduction

Partial or complete edentulism is still a common manifestation in all populations. One of the economic treatment options for teeth loss is the placement of partial or complete removable dentures. Polymers such as poly(methyl) methacrylate (PMMA) is a versatile material that is widely used in dentistry for the making of removable prostheses. The nature of these polymers being porous with rough or irregular surfaces makes them a suitable environment for microorganism adhesion [1]. Generally, *Candida albicans* (*C. albicans*) can be present in normal oral flora without producing any clinical symptoms or pathological changes [2, 3]. However, the distribution or count of oral flora might change depending on the individual's age and general and oral health [2, 3]. Also, the presence of intraoral appliances like acrylic dentures acts as a reservoir for microorganisms [4]. The use of a denture creates an environment characterized by low oxygen level and acidic pH suitable for *C. albicans* growth; this might be facilitated by reduced salivary flow under the intimately adapted denture base [2, 3].

Previously, studies reported that adherence of *C. albicans* to oral surfaces may precede colonization and infection [5, 6], and the formation of biofilm is crucial to develop denture-induced stomatitis (DS) [7]. Between the vast number of *Candida* species, *C. albicans* is considered the main causative pathogens for DS [1, 8]. The first step in colonization and initiation of DS involves the adherence of *C. albicans* to denture [6, 9] and host mucosal surfaces [10]. Among the factors that may affect *Candida* adherence to surfaces are the material (substrate) [11], surface topography or roughness [11, 12], presence of saliva [11], hydrophobicity [13], and other factors such as diet, presence of other microorganisms, and culture conditions [14]. Rough surfaces may be considered ideal for *C. albicans* adhesion since they provide larger area for adhesion and protected sites for colony formation away from the oral cleaning mechanisms [15]. However, the talk about the effect of surface roughness on microorganism adhesion especially yeast was not conclusive. A number of studies reported an increase in the yeast count with rougher surfaces [11, 16], while others stated that there is no effect of surface topography on *C. albicans* adherence and count [14, 17]. Besides the surface characteristics, the method of acrylic polymerization [4], oral and denture hygiene practices by patients [8], and dietary uptake and salivary flow [1] were all found to have an influence on debris, biofilm formation, and adhesion.

Proper home care and meticulous oral hygiene regimens are important for biofilm removal [10]. Mechanical or chemical plaque control techniques were found to cause resolution of DS [1, 18]. Suggestions of soaking dentures in sodium hypochlorite or chlorhexidine mouthwashes were recommended to remove plaque off from denture surfaces [1]; however, this protocol should not be a daily routine as this solution may be objectionable due to residual taste or odor, in addition to the bleaching and crazing effect on acrylic resin [2].

Few attempts were made to reduce *C. albicans* adhesion and further colonization to denture surfaces other than the conventional techniques. These include surface coating with different chemicals such as silane and chlorhexidine [10], bonding agent and 2-octyl cyanoacrylate adhesive [19], or incorporation of antifungal agents such as nystatin or amphotericin within acrylic resin [10]. Other studies suggested that the addition of tea tree essential oil [20] or henna powder [21] proved to limit the growth of *C. albicans*.

The advancement of medicine in recent years was directed at the use of nanotechnology to improve the delivery of medications compared with traditional systems. The ability of nanoparticles to penetrate into cell walls and membranes increases its effect and widens its range of use [22]. Because of nanoparticle nature and size being similar to biological and molecular structures, and their ability to work as drug carriers, they have been used to target pathogenic microorganisms [22]. However, and as any other material, nanoparticles could act as a two-sided agent with some adverse effects, necessitating careful determination of the proper therapeutic dose [22]. It has been found that the effect of these nanoparticles is also dependent on exposure time, temperature, particle size and shape, chemistry of the surface, and type of targeted cell [23]. Metal nanoparticles have been known for their antibacterial, antiviral, and antifungal properties. Many of them have been incorporated in dental polymers and composite adhesives to create antimicrobial nanocomposites capable of limiting the microbial and fungal growth and adhesion. Of these nanoparticles are nanosilver [24, 25], nanotitania and ferrite [26], nanodiamond [27], and nanozirconia [9].

Zirconium dioxide nanoparticles (nano-ZrO_2_) have received attention due to their favorable properties being biologically compatible with acceptable color [28]. Mechanical properties of PMMA/ZrO_2_ nanocomposite were found to be better than those of pure acrylic resin, including surface hardness, porosity reduction, and impact [29], flexural [25], and tensile strengths [30].

Studies on different fillers like glass ionomer showed that the presence of fillers will lower biomass volume, biofilm thickness, metabolic activities, and hyphal length [31]. Xu and colleagues looked at the effect of sustained nano-silver release on bacteria and found that it has an inhibitory effect on bacteria, which will in turn affect the biofilm and plaque formation [32]. Only a limited number of studies were carried out to assess the antifungal effect of nano-ZrO_2_ incorporated within denture base acrylic resin. Among these is a study by Gad et al. who looked at the antifungal effect of nano-ZrO_2_ addition to cold cure repair acrylic resin and found promising results represented by lower *C. albicans* adhesion [9]. Others like Gowri et al. reported the good antibacterial and antifungal capability of zirconia powder on its own or when used as a modifier of cotton fibers [33].

Nevertheless, good antimicrobial properties of nano-ZrO_2_-modified PMMA must also be accompanied by improved mechanical properties. Gad et al. reported higher surface hardness with 1–5% nano-ZrO_2_ [34]. However, the accompanying increase in surface roughness and its effect on *Candida* adhesion was not studied. Similarly, Gad et al. in two studies reported 2–5% nano-ZrO_2_ produced higher repair transverse strength [35], while 2.5–7.5% nano-ZrO_2_ resulted in improved tensile strength and lower translucency [30]. Ihab and Moudhaffar concluded that 2–5% nano-ZrO_2_ improved the impact and transverse strengths, while 7% loading had a negative effect [36]. Also, Zidan et al. reported better flexural strength, modulus, and surface hardness values at 3% nano-ZrO_2_ [37]. The lack of reported biological effects of different concentrations of nano-ZrO_2_ in relation to the tested mechanical properties eluded the authors to investigate a range of nano-ZrO_2_ addition to PMMA on surface roughness and *C. albicans* adhesion. The first null hypothesis was different loading ratios of nano-ZrO_2_ particles will not affect the surface roughness of the polymerized acrylic specimens. The second null hypothesis was different addition levels of nano-ZrO_2_ particles will not affect the *C. albicans* count.

## 2. Materials and Methods

### 2.1. Silanization of Nano-ZrO_2_ Particles

Nano-ZrO_2_ (99.9% purity, average size 40 ± 2 nm, surface area 9 ± 2 m^2^/g; Shanghai Richem International Co., Ltd., Shanghai, China) was silanized using 3-(trimethoxysilyl)propyl methacrylate (TMSPM; Shanghai Richem International Co., Ltd., Shanghai, China) in a manner similar to that described by Gad et al. [30] to obtain surface-treated nano-ZrO_2_ particles. This process improves the adhesion between the resin matrix and nano-ZrO_2_. The silanized nano-ZrO_2_ was weighed using a digital balance (S-234; Denver Instrument, Gottingen, Germany) and mixed with PMMA powder at three different concentrations (2.5wt%, 5.0wt%, and 7.5wt%). The mixture was stirred for 30 min to ensure homogeneity.

### 2.2. Acrylic Disc Preparation

A total of 20 discs (15 × 2 mm) were conventionally fabricated of heat-polymerized denture base acrylic resin (Major Base.20; Major Prodotti Dentari SPA, Moncalieri, Italy) in split metal flask. The specimens were separated into 4 groups (*n* = 5) according to the reinforcing filler concentration (0% without additives “control”), 2.5wt% (Z2.5), 5.0wt% (Z5.0), and 7.5wt% (Z7.5). The specimens were made in the conventional way of denture fabrication as prescribed in a previous study [9].

After polymerization, flasks were cooled and specimens were extracted from the stone, finished using carbide bur (HM251FX-040-HP; Meisinger, Centennial, CO, USA), and polished using polishing cloth (TexMet C10in, 42-3210; Buehler GmbH, Düsseldorf, Germany) and suspension (0.05 *μ*m, Master Prep, polishing suspension; Buehler GmbH) with the help of a mechanical polisher (MetaServ 250 grinder-polisher; Buehler, Lake Bluff, IL) at 250 rpm for 2 minutes under wet conditions. Specimens were kept in distilled water at 37°C for 1 week prior to testing to ensure the release of unreacted monomer and avoid the negative effects on cell viability [25].

### 2.3. Surface Roughness

A noncontact optical interferometric profilometer (Contour Gt-K1 optical profiler; Bruker Nano, Inc., Tucson, AZ) was used to record the surface roughness (*R*_*a*_) of the specimens. Each specimen was evaluated at three locations, and the values were averaged to get the final specimen roughness value in *μ*m.

### 2.4. Biofilm Assay of *C. albicans*

In advance to biofilm formation, *C. albicans* (ATCC 10231) from glycerol stocks were cultured on Sabouraud dextrose plates (SDB-Acumedica Co., Manufacturers, Inc.) at 30°C for 48 hours. Single isolated fresh colonies were inoculated overnight in Sabouraud dextrose broth at 30°C followed by shaking and then standardized using a spectrophotometer to 1 × 10^7^ cells/mL. Acrylic specimens were sterilized with 70% alcohol, ultrasonicated in sterilized distilled water for 20 minutes, and then exposed to UV light at room temperature for 30 min [38].

Each acrylic disc was immersed in 200 *μ*L standardized fungal broth and incubated at 37°C for 48 hours aerobically to allow biofilm formation. After incubation, specimens were gently rinsed two times by phosphate buffer saline (PBS) to eliminate loose microorganisms; then, they were placed within PBS-containing tubes in an ultrasonic bath. Adherent cells were counted using the direct culture method (colony-forming unit (CFU)), by streaking diluted suspension on agar media and incubating aerobically for 24 hours at 37°C [39]. A counting marker (Colony counter “SP Scienceware, Bel-Art Products”) was used to tally the *C. albicans* colonies. Tests were repeated under the same experimental conditions three times and in different days.

### 2.5. Scanning Electron Microscopy (SEM)

**Figure 1:**
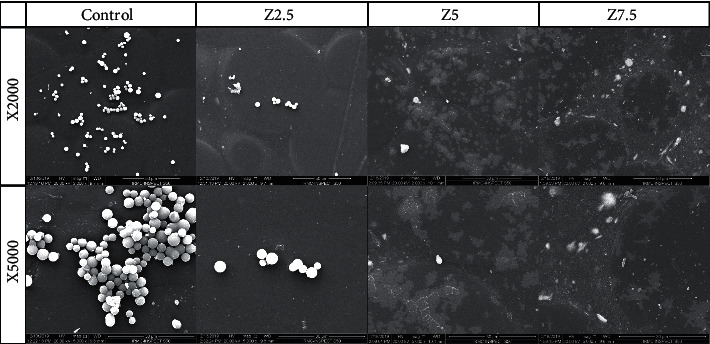
SEM images of acrylic specimens at two magnifications: x2000 (top row) and x5000 (bottom row). Columns from left to right are showing control, 2.5% nanozirconia-modified specimen, 5.0% nanozirconia-modified specimen, and 7.5% nanozirconia-modified specimen, respectively.

Scanning electron microscopy (SEM) is a useful tool to study the morphology and structure of the biological objects. To observe the morphological characteristic of biofilm, the specimen discs were chemically fixed with 2.5% glutaraldehyde solution at room temperature and then dehydrated using graded ethanol solutions. The specimens were then mounted on metallic stubs and sputter gold coated (Quorum, Q150R ES, UK) to overcome the nonconductive nature of the acrylic and biological objects (*C. albicans* cells). The mounted acrylic discs were inspected using an SEM instrument (Inspect S50; FEI, Czech Republic) at 20 kV. The representative electronic images of the control and test (reinforced) specimens are displayed in Figure 1 at two magnifications (×2000 and ×5000).

### 2.6. Statistical Analysis

IBM SPSS Statistics 23 (IBM Corp., Armonk, NY) was used for all statistical analyses. Insignificant results were found with the Shapiro–Wilk test, suggesting normally distributed data. Arithmetic means and standard deviations (SD) for studied variables were calculated. ANOVA was used to check overall significance followed by Tukey's post hoc test for pairwise comparisons. The level of statistical significance for all tests was set at *α* = 0.05.

## 3. Results

The means and standard deviations (SD) of surface roughness (*μ*m) and *C. albicans* adhesion (CFU/mL) are summarized in Table 1. ANOVA results for surface roughness (*R*_*a*_) revealed significant differences between the different groups of acrylic resin (*P*=0.01). Tukey's post hoc test was run to compare the surface roughness values between each pair of specimens, where the control (unmodified) acrylic resin specimens were significantly different from all other reinforced groups with the lowest recorded surface roughness value (0.027 ± 0.004 *μ*m). In between nano-ZrO_2_-reinforced groups (Z2.5, Z5.0, and Z7.5), there were no significant differences among the groups (*P* > 0.05) with Z5.0 showing the lowest surface roughness value (0.040 ± 0.006 *μ*m) and Z7.5 showing the highest surface roughness value (0.042 ± 0.004 *μ*m).

The means and SD of *C. albicans* adhesion (CFU/mL) are summarized in Table 1. ANOVA results were not found to be significant between the different groups (*P*=0.15). The control resin specimens recorded the highest *Candida* count value (1146.4 ± 703.0 CFU/mL) with a gradual decrease in *Candida* count as the nano-ZrO_2_ concentration increased. The Z7.5 group showed the lowest *Candida* count (498.4 ± 227.6 CFU/mL).

**Figure 2:**
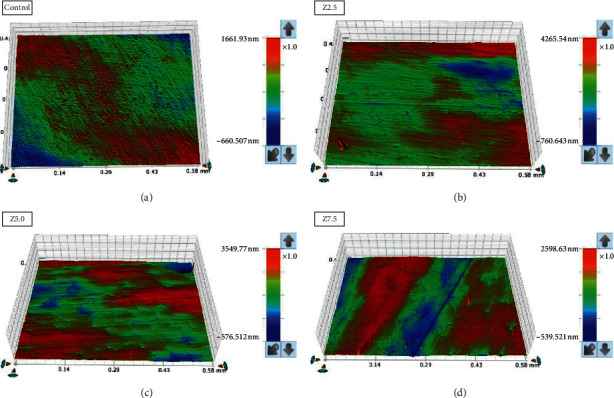
Images of surface roughness profile of specimens.

The surface morphological features of the control and reinforced acrylic specimens (Z2.5, Z5.0, and Z7.5) are represented by Figure 1. SEM analysis revealed that the surface morphology of the specimens is smooth in general even for nano-ZrO_2_-loaded specimens. The clusters of embedded nano-ZrO_2_ particles appeared in the reinforced specimens in the form of white patches, especially for Z5.0 and Z7.5 specimens. Regarding biofilm formation, a large number of *Candida* cells were found on the surface of the control specimen, whereby the *Candida* cells were randomly attached to the surface in the form of colonies and groups. On the contrary, no or fewer *Candida* cells were observed for the reinforced specimens; the number of attached cells is decreased as the nano-ZrO_2_ filler content increased. In addition, representative surface roughness profile images of the four groups are shown in Figure 2. The red color indicates high peaks, and blue represents deep valleys with a range of colors in between showing the intermediate heights.

## 4. Discussion

A number of studies carried out in the past [10, 19–21, 24–26] have tried to investigate different techniques to overcome DS through the use of surface coating, immersion in denture cleansers, or antimicrobial filler incorporation. The aim of the studies was to completely eliminate or at least reduce the adhesion of the causative microorganism; *C. albicans* through nanoparticle incorporation.

In this study, the intention was to evaluate the effect of nano-ZrO_2_ particle addition to heat-polymerized PMAA on the surface roughness and *C. albicans* biofilm. Based on the findings of this study, the first null hypothesis was rejected as the surface roughness changed significantly with the addition of nano-ZrO_2_ compared with unmodified specimens, while the second null hypothesis was accepted where different levels of nano-ZrO_2_ addition did not significantly affect the *Candida* count.

The literature has a number of studies documenting the improvement of multiple mechanical and physical properties of nano-ZrO_2_-modified PMMA at 2–7.5%. However, these studies did not evaluate the antimicrobial effects of these additions. Therefore, this study utilized these loading proportions (2.5, 5.0, and 7.5%) for PMMA modification and further evaluation.

With regard to surface roughness, adding nano-ZrO_2_ particles to the resin specimens caused a significant increase in roughness compared with the control. Similar findings were reported by a previous study [34] where surface roughness was directly related to the loading level of nano-ZrO_2_. On the contrary, another study [25] denied any change in surface roughness for nano-ZrO_2_-modified acrylic compared with control. However, in that study, the maximum ratio of nano-ZrO_2_ used was 1.5%, which might be the reason for insignificant roughness change. Similarly, Ihab and Moudhaffar [36] reported no change in surface morphology with higher concentrations of filler (2–7%). The discrepancies reported may be due to limitations of the contact-measuring tool used in that study and its inability to accurately read surface irregularities smaller than the diameter of its measuring tip.

The literature documented that surface roughness of the modified acrylic specimens depends to some degree on the size, amount, and distribution of the fillers within the matrix [40]. The nanoscaled particles have the ability to fill the interpolymeric spaces and lower the number of surface pores [29], which in return could lower the number of harbored surface microorganisms. Additionally, silanization is known to improve nanoparticle dispersion within the resin matrix [29, 36]. On the other hand, excess amount of nanoparticles (higher concentrations) may cause aggregation, cluster formations, and loss of homogeneity [34], which might overcome the dispersing effect of the silane coupling agent and cause agglomeration. If these clusters are formed at the specimen surface, they may cause an increase in the roughness as might have happened in this study with all loading ratios (2.5–7.5%). Additionally, because nano-ZrO_2_ is known for its high hardness [30], the authors of this study believe that polishing the specimens practically grinds away the soft resin matrix and expose the harder nano-ZrO_2_ particles underneath, which might be another explanation for the increase in surface roughness seen in this study.

It is well documented in the literature that microorganisms favor rougher surfaces [6, 11, 12, 40] where they can find more sites for attachment and colonization. Thus, the rougher the surface, the higher the chance for more colonization. Nevertheless, the roughness values reported for all groups in this study were well below the clinically recommended surface roughness value for denture base acrylic resin (0.2 *μ*m) [41].

The results of this study indicated that different levels of nano-ZrO_2_ particles added to heat-polymerized acrylic resin had the ability to decreased the *Candida* count; however, this reduction was insignificant. Similarly, a study by Mangal et al. [27] reported specimens reinforced with 5wt% nano-ZrO_2_ having the highest *Candida* number and the thickest biofilm compared with the negative control and nanodiamond-reinforced specimens. Those findings were slightly different from what was reported previously in the literature. Gowri et al. [33] reported the antifungal action of nano-ZrO_2_ particles against *C. albicans* and *A. Niger*. Others reported a significant reduction in *Candida* count after incorporating as low as 0.5wt% [25] and up to 7.5wt% [9] nano-ZrO_2_ within the PMMA resin. However, the differences in the results may be due to the difference of materials (heat-polymerized vs. autopolymerized acrylic resin [9]) or different fabrication techniques (single-layer acrylic specimens vs. double-layer acrylic specimens [25]).

The nonsignificant reduction in the yeast count in this study may be attributed to the increase in surface roughness seen with the modified groups, where hideout crannies and crevasses are more prominent that will create great shelter areas for the *Candida* [11, 12].

With a close look at the results of the current study, it can be noticed that there was a reduction in the number of adherent cells with the modified specimens, and the reduction was found to be concentration dependent regardless of the surface roughness value. It is believed that the nanoparticles will assume one of the following mechanisms of action to fight *Candida*; they will generate reactive oxygen species, conjugate with the cell membrane, disrupt cell wall/membrane activity, or release metal ions [41]. The literature reported that ZrO_2_ has the ability to interfere with the cell viable activities and cause deformation of hyphae [9, 33]. Based on the results of the surface roughness and the reduction in the *Candida* count, it can be suggested that nano-ZrO_2_ particles have some degree of antimicrobial effect that was able to overcome the increase in the roughness.

SEM analysis showed smooth background of pure and 2.5% nano-ZrO_2_-reinforced specimens with absence of cluster formation on the surface and large number of *Candida* colonies, indicating minimal effect of low filler concentrations on surface roughness and *Candida* adhesion. As the filler load increased (5.0–7.5%), nano-ZrO_2_ clusters were more apparent at the surface. This suggests the effect of direct contact between *Candida* cells and nanoparticles in reducing the number of microorganisms [40, 42].

Overall, PMMA/ZrO_2_ nanocomposite denture base had higher surface roughness than pure acrylic. Nevertheless, roughness was still within clinically acceptable value [41]. Regardless of the insignificant decrease in *Candida* count, nano-ZrO_2_ has proven to be an adequate mechanical enhancer of the modified acrylic resin according to previous literature [25, 29, 30, 34, 36] and therefore could be used as an effective additive to reduce *Candida* adhesion.

According to the findings of this study, surface roughness increased while *C. albicans* count decreased, in contrary to the aforementioned correlation between the substrate roughness and *C. albicans* count. These findings necessitate further investigations to ascertain the mechanism of action of nanoparticles with extensive evaluation of surface properties including wettability and hardness in order to answer the following question: is this reduction in C*andida* count attributed to the activity of nano-ZrO_2_, the effect on surface properties, or both?

It is worth mentioning that this study had few limitations that must be taken in consideration when interpreting the results. Among these limitations are the low number of specimens in each group, the use of only one type of heat-polymerized acrylic resin, and the flat surface of the specimens, which did not mimic the actual denture anatomy completely but would allow for comparison with previous reports. The authors recommend further studies testing the use of different acrylic resins, different nanofillers, and multiple ratios of loading in addition to clinical experiment where there is a vast number of microorganisms and more accurate representation of the denture shape and surrounding environment. Additionally, further studies could include XTT assay besides what the authors used in this study: culture methods and SEM.

## 5. Conclusions

Within the limitations of this study, it could be concluded that

1. Nano-ZrO_2_ addition to PMMA denture base resulted in surface roughness increase. However, this increase was within the clinically acceptable value.
2. The addition of nano-ZrO_2_ particles to PMMA produced insignificant reduction in *C. albicans* count, and the reduction was concentration dependent.

## Figures and Tables

**Table 1:** Mean (SD) and statistical results of ANOVA and Tukey's post hoc tests for surface roughness (*μ*m) and *Candida* count (CFU/mL) for pure and modified acrylic resin specimens.

Group	Surface roughness (*μ*m)		*Candida* count (CFU/mL)	
	Mean (SD)	*P* value	Mean (SD)	*P* value
Control	0.027 (0.004)	0.01^*∗*^	1146.4 (703.0)	0.15
Z2.5	0.041 (0.006)^a^		634.75 (391.4)	
Z5.0	0.040 (0.011)^a^		589.5 (238.2)	
Z7.5	0.042 (0.004)^a^		498.4 (227.6)	

Note: the symbol “^*∗*^” indicates significant *P* value (ANOVA test). Similar small superscripted letter indicates no significant difference between the groups (*P* > 0.05).

## Data Availability

The data that support the findings of this study are available upon request from the corresponding author.
